# Profiles of Recovery from Mood and Anxiety Disorders: A Person-Centered Exploration of People's Engagement in Self-Management

**DOI:** 10.3389/fpsyg.2016.00584

**Published:** 2016-04-26

**Authors:** Simon Coulombe, Stephanie Radziszewski, Sophie Meunier, Hélène Provencher, Catherine Hudon, Pasquale Roberge, Martin D. Provencher, Janie Houle

**Affiliations:** ^1^Department of Psychology, Université du Québec à Montréal, MontréalQC, Canada; ^2^Faculty of Nursing, Université Laval, Québec CityQC, Canada; ^3^Department of Family Medicine and Emergency Medicine, Université de SherbrookeSherbrooke, QC, Canada; ^4^School of Psychology, Université Laval, Québec CityQC, Canada; ^5^Research Centre, Institut universitaire en santé mentale de Montréal, MontréalQC, Canada

**Keywords:** self-management, recovery, mood and anxiety disorders, person-centered approach, health engagement, positive mental health

## Abstract

**Context:** A shift toward person-centered care has been occurring in services provided to people with mood and anxiety disorders. Recovery is recognized as encompassing personal aspects in addition to clinical ones. Guidelines now recommend supporting people's engagement in self-management as a complementary recovery avenue. Yet the literature lacks evidence on how individualized combinations of self-management strategies used by people relate to their clinical and personal recovery indicators.

**Objectives:** The aims of this study were to identify profiles underlying mental health recovery, describe the characteristics of participants corresponding to each profile, and examine the associations of profiles with criterion variables.

**Method:** 149 people recovering from anxiety, depressive, or bipolar disorders completed questionnaires on self-management, clinical recovery (symptom severity), personal recovery (positive mental health), and criterion variables (personal goal appraisal, social participation, self-care abilities, coping).

**Results:** Latent profile analysis (LPA) revealed three profiles. The *Floundering* profile included participants who rarely used self-management strategies and had moderately severe symptoms and the lowest positive mental health. The *Flourishing* profile was characterized by frequent use of self-empowerment strategies, the least severe symptoms, and the highest positive mental health. Participants in the *Struggling* profile engaged actively in several self-management strategies focused on symptom reduction and healthy lifestyle. They concomitantly reported high symptom severity and moderately high positive mental health. The study revealed that *Floundering* was associated with higher probabilities of being a man, being single, and having a low income. People in the *Flourishing* profile had the most favorable scores on criterion variables, supporting the profiles' construct validity.

**Discussion:** The mixed portrait of *Struggling* participants on recovery indicators suggests the relationship between health engagement and recovery is more intricate than anticipated. Practitioners should strive for a holistic understanding of their clients' self-management strategies and recovery indicators to provide support personalized to their profile. While people presenting risk factors would benefit from person-centered support, societal efforts are needed in the long term to reduce global health inequalities. The integration of constructs from diverse fields (patient-centered care, chronic illness, positive psychology) and the use of person-oriented analysis yielded new insights into people's engagement in their health and well-being.

## Introduction

Contemporary mental health services are more person-centered^1^ than they used to be (Mechanic, 2007). Mental health providers increasingly seek to support people's engagement in their idiosyncratic recovery process rather than prescribing a rigid treatment plan (Corrigan, 2015). As an overarching philosophy behind person-centered care (Storm and Edwards, 2013), the notion of recovery orients the services offered to people living with mental disorders in several countries, such as the US (President's New Freedom Commission on Mental Health, 2003), England (National Institute for Mental Health in England, 2005), New Zealand (Mental Health Commission, 2012), and Canada (Mental Health Commission of Canada, 2009). From a clinical approach, recovery refers to the reduction of symptoms below the clinical threshold (e.g., Frank et al., 1991). In contrast, in a person-centered approach, recovery refers to “a movement toward health and meaning rather than avoidance of symptoms” (Clarke et al., 2012, p. 303). Self-management (i.e., daily actions a person takes to manage symptoms and well-being) has been proposed as a crucial pathway to recovery from mental disorders (Slade, 2009). Building on people's engagement in their own well-being and health (Graffigna et al., 2014), supporting self-management appears to be an exemplary person-centered practice. However, the notion of self-management mainly derives from the chronic disease literature (Lorig and Holman, 2003; Sterling et al., 2010), and its application in mental health recovery research is still limited (see Mueser et al., 2002, for a review in the mental health field). The aim of this study was to examine recovery from mood and anxiety disorders by focusing on the person and his/her active role. The present study constitutes a first exploration of individual profiles underlying mental health recovery. It highlights different combinations of self-management strategies used by people in relation to recovery indicators. To this end, innovative person-oriented analyses were conducted to discern how self-management and recovery are related at the person level, in contrast to traditional variable-oriented analyses that consider relationships between variables across whole groups of participants (Meyer et al., 2013).

### Recovery from mood and anxiety disorders

Mood and anxiety disorders are among the most prevalent mental disorders in the world (Kessler et al., 2005, 2007). In the US, lifetime prevalence has recently been estimated at 17.5% for any mood disorder (major depressive and bipolar disorders) and 31.6% for any anxiety disorder (panic, generalized anxiety, agoraphobia, social phobia, specific phobia, separation anxiety, post-traumatic stress, obsessive-compulsive disorders) (Kessler et al., 2012). In Canada, an estimated 11.6% (point prevalence) of the adult population reported having a mood or anxiety disorder (Public Health Agency of Canada, 2015). Mood and anxiety disorders are often recurrent. The estimated cumulative recurrence rate for major depressive disorder has been estimated at 42.0% at 20 years after remission (Hardeveld et al., 2013). Indicative of chronicity, in a study of people living with anxiety disorders, the average time spent in an illness episode represented over 70% of the 12-year study course (Bruce et al., 2005). Mood and anxiety disorders are also highly comorbid. For example, a study with a large nationally representative sample in the Netherlands estimated (12-month prevalence) that 54.3% of people with a mood disorder also had an anxiety disorder, and 33.4% of those with an anxiety disorder also had a mood disorder (de Graaf et al., 2002). Given the comorbidity and similitudes between these disorders, “it is sensible to consider them as a single group,” as argued by the International Society for Affective Disorders^2^, the leading international scientific society in that field.

Mental health recovery from mood and anxiety disorders has usually been defined using a *clinical* approach, i.e., as a reduction of clinical symptoms to below a threshold for a certain period of time, following Frank et al.'s (1991) definition (see review from Fava et al., 2007). However, this pathogenic approach is now being deemed too limited in comparison with how mental health consumers themselves define recovery (Zimmerman et al., 2006; Johnson et al., 2009; McEvoy et al., 2012). From their perspective, recovery is better defined as “a deeply personal, unique process of changing one's attitudes, values, feelings, goals, skills, and/or roles. It is a way of living a satisfying, hopeful, and contributing life even with limitations caused by illness” (Anthony, 1993, p. 527). This *personal* approach to recovery is concordant with the recent field of positive psychology that aims to cultivate human strengths, well-being, and dimensions that make life worth living (Seligman and Csikszentmihalyi, 2000; see Provencher and Keyes, 2010, 2011, 2013).

Personal and clinical approaches to recovery have mainly been examined in distinct streams of research. However, Whitley and Drake (2010) recently proposed a theoretical conceptualization of recovery that encompasses both clinical and personal aspects. Their model postulates five recovery dimensions: clinical (e.g., reduction and control of symptoms), existential (e.g., emotional and spiritual well-being), functional (e.g., employment and education), physical (e.g., diet and exercise), and social (e.g., social support and community integration). Although Whitley and Drake (2010) suggest a list of several measurable outcomes that could be used to explore these dimensions of recovery, to our knowledge their comprehensive assessment has yet to be fully operationalized.

Provencher and Keyes (2010, 2011, 2013) also proposed a comprehensive model: the Complete Mental Health Recovery model. Based on this model, recovery should be assessed on two indicators. The first is the experience of restoration from mental illness symptoms; the second is the experience of optimization of positive mental health. The first indicator mostly pertains to the clinical recovery approach, while the second mostly relates to themes from the personal recovery approach (Slade, 2010). Positive mental health is defined as a syndrome composed of several manifestations of well-being (Keyes, 2002), at the emotional (e.g., interest, satisfaction), psychological (e.g., purpose in life, personal growth), and social levels (e.g., social contribution, social integration). Provencher and Keyes' model is based on several psychometric studies using large non-clinical samples showing mental illness and positive mental health to be two coexistent dimensions, and not merely the two ends of a single dimension (Keyes and Lopez, 2002; Keyes, 2005; Keyes et al., 2008; Westerhof and Keyes, 2010).

Formed by the intersection of these two dimensions, Provencher and Keyes (2010, 2011, 2013) model proposes different states of recovery. In partly recovered states, the person shows low symptoms^3^ concomitantly with low positive mental health (state labeled as *languishing* by Keyes and Lopez, 2002) or high symptoms and high positive mental health (labeled as *struggling* with life). In the completely recovered state, the person shows both low symptoms and high positive mental health (labeled as *flourishing*). In the opposite state, the person is non-recovered on both aspects (labeled as *floundering*). The model also proposes two more states, in which people have a moderate level of positive mental health but are either recovered or not from their symptoms. According to their situation in terms of recovery indicators (symptom severity and positive mental health), individuals are expected to fall into one of these states. However, this classification has never been explored in clinical samples of people with mood and anxiety disorders.

### Self-management in mental health recovery

Exploring recovery from a person-centered perspective necessitates considering what people actually do in their pathway toward recovery. Self-management refers to actions people implement day-to-day to manage their symptoms, prevent recurrence, and optimize well-being (Lorig and Holman, 2003). Self-management harnesses people's sense of agency, responsibility, empowerment, and motivation to get better (Barlow et al., 2005; Slade, 2009). Self-management support is now recommended in clinical guidelines for mood and anxiety disorders (Swinson et al., 2006; Patten et al., 2009; National Institute for Health and Care Excellence, 2014). Supporting self-management is intended to complement, not to replace, standard psychological, and pharmacological treatments (Fournier et al., 2012). It is a useful approach to complement such evidence-based treatments, which, although efficient, are limited by the fact that not all people respond positively to antidepressants or psychotherapy (Bystritsky, 2006; Lanouette and Stein, 2010; Berlim et al., 2015), and that several of them relapse (Boland and Keller, 2009; Boschen et al., 2009) or must deal with incapacitating residual symptoms (Fava et al., 2007; Kaya et al., 2007).

The value of self-management for coping with physical chronic illness such as diabetes and asthma has been well established (Barlow et al., 2002). This is in line with a prolific stream of theoretical and empirical work in medicine on the broader concepts of engagement and active involvement in one's own health and care (see review from Menichetti et al., 2014). While similar to self-management, health engagement has recently been proposed as an umbrella term (Graffigna et al., 2015b) representing a multidimensional process that includes not only behaviors (Gruman et al., 2010) but also the person's cognitions and emotions regarding his/her health (Graffigna et al., 2014). These dimensions can be considered at different levels of the person's systemic context (e.g., individual, organizational, societal; Carman et al., 2013).

In contrast, self-management is more specific, as it focuses on strategies (behaviors) that the person enacts, considered as one positive outcome of the engagement process (see review from Graffigna et al., 2015b), while patient activation focuses on the knowledge, skills, and confidence for performing such strategies (Hibbard and Mahoney, 2010). Notions of engagement and activation have received only limited attention in the mental health field or in psychology (Kukla et al., 2013; Menichetti et al., 2014; Sacks et al., 2014; Moljord et al., 2015). Similarly, research and interventions on self-management are less frequent in the context of mental illness than in medicine (Cook et al., 2009; Lorig et al., 2014). In the present article, while the center of attention is self-management, the findings also have the potential to contribute to the incipient knowledge base on the application of these related concepts to the field of mental health.

Self-management strategies implemented by people with mood and anxiety disorders have rarely been studied, with the exception of a few recent qualitative studies (Murray et al., 2011; van Grieken et al., 2014, 2015; Chambers et al., 2015; Villaggi et al., 2015). Participants have reported a wide variety of strategies focused on reducing and preventing symptoms (e.g., mood monitoring, obtaining mental health services), as well as other strategies to promote positive mental health (e.g., meditating, socializing). In the study from Villaggi et al. (2015), participants with depressive, bipolar, and anxiety disorders reported overall similar strategies, suggesting that a transdiagnostic approach to self-management is appropriate.

Based on this qualitative study (Villaggi et al., 2015), our research team developed the Mental Health Self-management Questionnaire (MHSQ), the first instrument to provide a quantitative indicator of the frequency with which people use a diversity of strategies (Coulombe et al., 2015). The validation study revealed three distinct types of self-management strategies: (a) clinical (getting help and using resources, e.g., taking medication, consulting a professional); (b) empowerment (building upon strengths and positive self-concept to gain control, e.g., acknowledging one's successes, arranging one's schedule around one's capabilities); and (c) vitality (having an active and healthy lifestyle, e.g., practicing sports, maintaining healthy eating habits).

In our cross-sectional validation study of the MHSQ (Coulombe et al., 2015), positive mental health was associated positively with empowerment and vitality strategies but unrelated to clinical ones. Depressive and anxiety symptom severity indicators were found to be negatively related to empowerment and vitality strategies. However, symptom severity was positively related to clinical self-management. This was interpreted as suggesting that participants with more severe symptoms may have focused on using clinical strategies, given their acute needs in that regard. Indeed, people with severe symptoms have been shown to be more likely to use health services (Hämäläinen et al., 2008), one of the so-called clinical strategies. In contrast, people with less severe symptoms may have been more likely to use empowerment and vitality strategies, since they probably had reached a different state of recovery and now faced the task of increasing their positive mental health (Provencher and Keyes, 2011). These interpretative hypotheses illustrate the need for further studies to disentangle the complex relationships between self-management and recovery indicators.

### The value of person-oriented statistical analysis

As people have been shown to use their personal “recipe” of self-management strategies (Chambers et al., 2015; Villaggi et al., 2015), it is important to go beyond the group level when exploring self-management and recovery. Given the variety of self-management strategies and possible situations in terms of recovery indicators (i.e., forming six different states according to Provencher and Keyes, 2010, 2011, 2013), it is pertinent to ask how these all vary together, and whether, across individuals, there are diverse profiles of interrelationships among these variables. Exploring such profiles quantitatively calls for person-oriented analyses. In contrast to the variable-oriented approach (e.g., correlational analysis), person-oriented analysis [e.g., cluster analysis, latent profile analysis (LPA)] allows variables to be related differently across the people in the sample (Meyer et al., 2013). The individual is seen as a system of variables that “can combine in various ways that have implications for how they are experienced and relate to other variables of interest” (Meyer et al., 2013, p. 191). Person-oriented analyses are intended to provide a holistic perspective, offering a richer source of information for person-centered services (Cloninger, 2013). One person-oriented analysis that is gaining in popularity is LPA, which provides a way to uncover unobserved (i.e., latent) profiles of participants showing distinctive patterns of interaction among continuous variables. In LPA, the number of profiles is selected based on the estimation and comparison of statistical models, allowing for more objectivity than other procedures, such as cluster analysis (DiStefano and Kamphaus, 2006; Pastor et al., 2007; Morin et al., 2011b).

Once these profiles underlying mental health recovery are identified, it is possible to explore the background characteristics (on clinical and sociodemographic variables) associated with each profile. Notably, although evidence is still scarce, people might show different profiles depending on their diagnoses. A recent study (Vermeulen-Smit et al., 2015) suggests that having a depressive or anxiety disorder is associated with lower probability of endorsing a healthy lifestyle (i.e., vitality self-management strategies), while this is not the case with bipolar disorder. Treatments currently in progress are another factor to consider. People with more severe symptoms could be more likely to receive mental health services (Hämäläinen et al., 2008) and concomitantly to display a profile characterized by the use of self-management strategies focused on symptoms (Coulombe et al., 2015).

Sociodemographic variables are also important. Recovery- and person-centered policies and research emphasize the importance of holistic approaches that take into account social determinants of health, such as gender, income, and marital status (Jayadevappa and Chhatre, 2011; Weisser et al., 2011; Commonwealth of Australia, 2013; Cloninger et al., 2014). Nevertheless, in their review, Weisser et al. (2011) concluded that recovery has mainly been studied as “an individual journey,” so the existent literature “falls short on an analysis of the role of gender and other social and structural inequities in mental health problems” (p. 6). For instance, because of their endorsement of traditional masculinity norms, men would probably use fewer self-management strategies, such as seeking professional help (Möller-Leimkühler, 2002). Also, being married is associated with increased adherence to health recommendations, possibly because of the social support offered by a life partner (e.g., Trivedi et al., 2008). Finally, being from a low-income background is associated with less health engagement (Greene and Hibbard, 2012), as there are economic barriers to self-management (Henderson et al., 2014). Despite the formative evidence, background factors have never been examined specifically in relation to self-management and to clinical and personal recovery.

As stated by Morin et al. (2011b, p. 61), “the advantages of LPA do not offset the need to assess the construct validity of the classification.” Profiles are considered valid to the extent that their pattern of association with criterion variables is consistent with theoretical expectations (Bauer and Curran, 2004; Morin et al., 2011b). Thus, the associations of recovery profiles with meaningful criterion variables need to be examined. In the present study, four were selected: personal goal appraisal, social participation, self-care abilities, and coping.

*Personal goals* constitute a pervasive theme in the recovery literature (Andresen et al., 2003). Empirical research has consistently related mental health indicators to positive appraisal of one's personal goals, in terms, for example, of how important they are or how effective one is at achieving them (Little, 2007). Negative goal appraisal has been related to depression, anxiety, and hypomania (Lecci et al., 1994; Meyer et al., 2004; Dickson et al., 2011). Getting and seizing opportunities for *social participation* have also been highlighted as important components in recovery (e.g., Noordsy et al., 2002; Onken et al., 2007; see Provencher and Keyes, 2011). For people in recovery, regaining some of their previous social roles and engaging in new ones can give meaning to their life (Mezzina et al., 2006). *Self-care abilities* refer to people's knowledge and competence concerning activities they need to perform for their health (Britz and Dunn, 2010; Seed and Torkelson, 2012). These are foundational skills for effective self-management. Similarly, the way people cope with illness has been related to psychological adjustment (Roesch and Weiner, 2001). In this context, *coping* refers to people's adaptive (e.g., planning, seeking support) and maladaptive (e.g., denial, substance use) efforts to deal with the stress associated with their disorder (Meyer, 2001; Roesch and Weiner, 2001).

Self-management and recovery indicators, being comprehensive variables, were chosen as the key parameters driving the profile exploration in the present study. In contrast, personal goal appraisal, social participation, self-care abilities, and coping are more specific notions. These are nevertheless interesting to consider as criterion variables, given their importance in recovery theories and findings. However, because the profiles have never been explored before, their precise nature is still unknown; thus it would be premature to propose specific hypotheses concerning their associations with the criterion variables (Morin et al., 2011b).

### Objectives

The aim of this study was to explore person-centered recovery profiles presented by individuals who reported having received a diagnosis of mood and anxiety disorders. The first objective was to identify and draw the general portrait of the distinct profiles concerning individuals' use of self-management strategies (clinical, empowerment, and vitality) and scores on recovery indicators (symptom severity and positive mental health). The second objective was to describe the profiles by exploring their associations with (a) the frequency of use of specific self-management strategies and (b) background characteristics (clinical and sociodemographic variables). The third objective was to verify the construct validity of the profiles by examining their pattern of association with criterion variables.

## Materials and methods

### Procedure

The present study was part of a larger research project to validate the (MHSQ, see Section Self-Management). Validation results have been published elsewhere (Coulombe et al., 2015). Using data from that study, the present paper is distinct by virtue of its different analytical strategy (person-oriented analysis) and its consideration of an array of variables (e.g., gender, low income, personal goals, etc.) that were not treated in the validation article. The study was approved by the institutional research ethics board for research involving human participants at Université du Québec à Montréal, Canada.

#### Recruitment

Thirteen community organizations in Quebec (Canada) and France were asked to send an email invitation to members of their mailing list and to advertise the study on their website. An invitation was also published in a Montreal (Canada) free newspaper. The invitation included a URL link for participants to complete the study online. After reading and consenting to an online consent form, participants answered self-reported preliminary questions to verify their eligibility. Participants had to be at least 18 years old; understand written French; have received a diagnosis of anxiety, depressive and/or bipolar disorder(s) at least 1 year prior to responding; and be in treatment or have been treated (with psychotherapy and/or pharmacotherapy) for the disorder(s). The “time since diagnosis” criterion was intended to ensure the person had had sufficient time to implement self-management strategies. Pregnant women or those who had given birth in the previous year were excluded, given that the recovery process is different in these situations (Hendrick et al., 2000). To prevent symptom exacerbation due to filling out the questionnaire, people scoring high on symptom measures (see Section Recovery Indicators) were excluded and presented with a list of available help resources. The same list was presented to all participants after questionnaire completion. The questionnaire was filled out on a secured online survey platform.

#### Participants

The final sample was composed of 149 participants. The detailed sample description has been published in the MHSQ validation paper (Coulombe et al., 2015). The majority of participants reported having been diagnosed with a depressive disorder (55.7%), while self-reported anxiety (36.9%), and bipolar (36.2%) disorders were less prevalent. In terms of comorbidity, around one-quarter (26.8%) reported having been diagnosed with more than one of these disorders. Based on the scores on the depression severity measure (see Section Recovery Indicators; Kroenke and Spitzer, 2002), at the time of the study, 34.2% of the participants reported moderate symptoms, 30.2% mild symptoms, and 35.6% less than mild symptoms. Based on the scores on the anxiety severity measure (Spitzer et al., 2006), 26.2% reported moderate symptoms, 26.8% mild symptoms, and 47.0% less than mild symptoms. The vast majority reported they had been undergoing pharmacotherapy (85.2%) in the past month, and less than half of the sample was currently undergoing psychotherapy (40.3%). Participants were mostly female (80.1%) and were on average 41.5 years old (*SD* = 12.2; from 18 to 71). Most reported being from Canada or having immigrated there (91.9%). The sample was very educated: 60.4% had a university degree, which is much higher than the Canadian figure (30.8%, Statistics Canada, 2013). The remaining participants either had a vocational (9.0%) or a college (pre-university) degree (21.5%), or a high school diploma or less (9.0%). About half the participants were married or had a life partner (47.9%) while the other half (52.1%) were single. As explained below (Section Background Characteristics), low-income status was calculated only for those from Canada; nearly one-quarter (23.0%) were living under the low-income threshold (Statistics Canada, 2015).

### Measures

The questionnaire included the validated French version of the following instruments.

#### Self-management

Self-management was measured using the MHSQ developed as part of the larger study (see **Table 5** for the complete item list). Items were created on the basis of qualitative interviews (Villaggi et al., 2015), and a multidisciplinary expert team helped reduce the number of items and improve wording. As reported in the validation paper (Coulombe et al., 2015), exploratory and confirmatory analyses of data collected from the present sample indicated the presence of three distinct subscales: (a) clinical (5 items, e.g., *I look for available resources to help me with my difficulties (websites, organizations, healthcare professionals, books, etc.); I participate in a support or help group to help me manage my difficulties*); (b) empowerment (9 items, e.g., *I take my capabilities into account when arranging my schedule; I congratulate myself for my successes, large and small*); and (c) vitality (4 items, e.g., *I do activities I like to maintain an active lifestyle; I engage in sport, physical activity*). For each item, participants were asked to indicate to what extent they had used the strategy during the two previous months, on a scale from 0 (*Never*) to 4 (*Very often*). Each subscale had adequate internal consistency: α = 0.69 for clinical, α = 0.81 for empowerment, and α = 0.75 for vitality.

#### Recovery indicators

Three recovery indicators were included, two measuring recovery from the clinical perspective (symptom severity) and one from the personal perspective (positive mental health).

The Patient Health Questionnaire 9 (PHQ-9; Kroenke and Spitzer, 2002) was used to assess severity of depressive symptoms. The PHQ-9 requires participants to rate to what extent they had experienced nine symptoms (e.g., *little interest or pleasure in doing things*) during the two previous weeks, on a 4-point frequency scale: 0 (*Not at all*), 1 (*Several days*), 2 (*More than half the days*), and 3 (*Nearly every day*). The Generalized Anxiety Disorder 7 (GAD-7; Spitzer et al., 2006) was used to assess severity of anxiety symptoms (e.g., *feeling nervous, anxious, or on edge*) on the same response scale. According to a systematic review (Kroenke et al., 2010), the PHQ-9 and GAD-7 have adequate sensitivity and specificity for detecting symptoms of depressive and anxiety disorders and monitoring their severity. Both scales had adequate internal consistency in the current study: α = 0.85 for PHQ-9 and α = 0.86 for GAD-7. Sums of scores for each scale were used as recovery indicators in the analyses, but also to verify eligibility, with participants presenting severe symptoms (PHQ-9 ≥ 20; GAD-7 ≥ 15; Kroenke and Spitzer, 2002; Spitzer et al., 2006) being excluded, as explained above. The Altman Self-Rating Mania Scale (ASRMS, Altman et al., 1997) was used to exclude participants who were in current mania (ASRMS ≥ 6; Altman et al., 1997).

The Mental Health Continuum–Short Form (MHC-SF; Keyes, 2002; Lamers et al., 2011; Salama-Younes, 2011) was used to assess the degree of participants' positive mental health in terms of their experience of 14 well-being manifestations, related to positive emotions (e.g., *feel* s*atisfied with your life*), psychological functioning (e.g., *feel that your life has a sense of direction or meaning to it*), and social functioning (e.g., *feel that you belonged to a community*) during the past month. Participants were required to answer on a 6-point frequency scale: 0 (*Never*), 1 (*Once or twice*), 2 (*About once a week*), 3 (*About two or three times a week*), 4 (*Almost every day*), and 5 (*Every day*). The MHC-SF has been shown to be valid and reliable in diverse samples (Keyes et al., 2008; Lamers et al., 2011) and has been successfully used in national surveys (e.g., Canadian Community Health Survey—Mental Health, Statistics Canada, 2012). Results from several studies with large samples (>1000) across the world show that MHC-SF scores are not simply the inverse of mental illness symptom indicators, as they measure two distinct factors that correlate negatively but only moderately (Keyes et al., 2008; Lamers et al., 2011; Petrillo et al., 2015). Internal consistency was satisfactory (α = 0.92) in the present study.

#### Background characteristics

In terms of clinical variables, diagnosis (depressive, anxiety and/or bipolar disorders) and ongoing treatments (undergoing psychotherapy and/or pharmacotherapy) were self-reported as part of the eligibility questions. The questionnaire also included a sociodemographic form including age, gender, education level, marital status, number of people in the household, and household income. Using the last two variables, each participant's status as living or not in a low-income household was determined based on the national cut-off depending on household size (Statistics Canada, 2015). For comparability purposes, only participants from Canada, who made up the vast majority of the sample, were included in the analysis pertaining to low income.

#### Criterion variables

Assessment of participants' personal goal appraisal was based on the Personal Project System Rating Scale (PSRS; Little, 1988; Pychyl and Little, 1998; Chambers, 2007), which was translated into French and adapted for the purposes of the present study. Participants were asked to appraise their goal system (presented as their current goals, activities, commitments, and projects considered on the whole) on a scale from 1 (*Not significant for me*) to 10 (*Very significant for me*) along six dimensions: meaningfulness, manageability, progress, support, stress (reversed), and enjoyment. Cronbach's alpha of the overall scale was satisfactory (α = 0.82).

Social participation was measured with the Social Participation Scale (Richard et al., 2009). The scale assessed to what extent participants had taken part in 10 social activities (e.g., visiting friends or family, shopping, volunteering) in the previous 6 months, on a 5-point frequency scale: 0 (*Never*), 1 (*Less than once a month*), 2 (*At least once a month*), 3 (*At least once a week*), and 4 (*Almost every day*). Internal consistency was satisfactory (α = 0.70).

The Therapeutic Self-Care Scale (Doran et al., 2002; Paradis, 2009) was used to assess participants' perceived self-care abilities. The 12 items were developed for patients living with physical illness, but are also pertinent in a mental health context. The scale measures knowledge and competence with regard to management of the disorder, such as understanding what needs to be done to address one's symptoms, being able to take one's medication (if applicable), etc. Answers were given on a 6-point Likert scale, from 0 (*Not at all*) to 5 (*Completely*). Cronbach's alpha was high (α = 0.86).

Use of coping strategies was measured with the Brief COPE (Carver, 1997; Muller and Spitz, 2003), in which participants indicated to what extent they had used 28 strategies to deal with the stress associated with their mental health problem, on a 4-point scale: 0 (*Not at all*), 1 (*A little bit*), 2 (*Moderately*), and 3 (*A lot*). Instead of using the instrument's 14 original subscales, four coping subscales were created to reduce the number of variables in the analysis, following the procedure used by Desbiens and Fillion (2007): emotional (venting and emotional support; α = 0.80), behavioral (active coping, planning, and instrumental support; α = 0.85), cognitive (acceptance, positive reframing, humor, and religion; α = 0.77), and avoidance (substance use, denial, behavioral disengagement, and self-distraction; α = 0.67).

### Analysis

As a preliminary analysis, bivariate correlations were examined between the main study variables. To achieve the first objective, LPA was then performed using the Robust Maximum Likelihood estimator (MLR) available in the Mplus software (Muthén and Muthén, 1998-2010) to identify latent profiles of participants (Morin et al., 2011b; Morin, 2016), based on participants' scores on the three subscales of self-management strategies and on the three recovery indicators. To ensure the analysis did not converge on a local solution, the estimation process aimed to replicate the solution, using 3000 sets of random starts and 100 iterations, and retaining the 100 best sets of starting values for final stage optimization^4^, following Morin's recommendation (2016). Models with increasing numbers of profiles were compared using a variety of statistical criteria. Lower values of the Akaike Information Criterion (AIC), Consistent AIC (CAIC), Bayesian Information Criterion (BIC) and sample-size adjusted BIC (SSA-BIC) indicated better fit. Tests comparing each model with the model having one less profile (Vuong-Lo-Mendell-Rubin Likelihood Ratio, VLMR; Adjusted Lo-Mendell-Rubin Likelihood Ratio, ALMR; and Bootstrap Likelihood Ratio Test, BLRT) were also considered: significant *p*-values for these tests indicated that the model with more profiles was more adequate. Models including profiles in which fewer than 5% of the participants are classified should be rejected (Hamza and Willoughby, 2013). Finally, although entropy (which varies from 0 to 1) cannot be used to identify the optimal number of latent profiles in the data, it provides useful information regarding the accuracy of the participants' classification into the various latent profiles, with higher levels being indicative of less classification error (Tein et al., 2013; Morin, 2016)

Once the number of profiles was selected, each profile's standardized means on the self-management subscales and recovery indicators were graphed and compared with the overall sample mean. The profiles were also compared to one another on these variables, by re-running the LPA in Mplus, but adding an auxiliary command named “auxiliary (e),” which provides equality of means tests across profiles. Introduction of variables using such a command does not have an impact on the nature of the profiles (Morin et al., 2011b). Using an auxiliary command has recently been presented as one of the best ways of studying the association between variables and latent profiles (Asparouhov and Muthén, 2014b; Feingold et al., 2014). It recognizes classification uncertainty, and thus each participant is correctly considered as having a degree of probability of being a member of every profile (Bolck et al., 2004; Morin, 2016). For pragmatic purposes, as an additional analysis that could facilitate interpretation for practitioners, we performed an analysis in which participants were classified into only one of the profiles based on their *Most Likely Latent Profile Membership*. Each self-management subscale and recovery indicator was dichotomized into high and low scores, using the documented clinical cut-off when available (for symptom severity) or, when not available, by splitting the variable at the nearest score above the overall mean. The distributions of high (vs. low) scores were then compared across profiles with chi-square using SPSS software. Despite the fact that this involves a certain loss of information compared to the auxiliary command, this supplementary analysis is particularly informative for transposing our results to clinical settings, in which practitioners will find useful to have a clear portrait of clients that would be assigned to each profile.

To achieve the second objective (part a), scores on individual items of the self-management questionnaire were compared across profiles. To do so, the LPA was re-run in Mplus, but this time variables corresponding to the individual self-management items were integrated using another auxiliary command, named BCH, designed for such purposes (Asparouhov and Muthén, 2014a). This tested equality of means across profiles for each self-management strategy.

To achieve the second objective (part b), associations between profiles and participants' background characteristics (clinical and sociodemographic variables) were examined by introducing these characteristics using the auxiliary BCH command for continuous variables (in our case, only the age variable) and another similar command, named DCAT, designed for categorical variables (all the variables other than age) (Asparouhov and Muthén, 2014a). The DCAT command provides a between-profile comparison of the estimated probability of each characteristic.

To achieve the third objective, associations between profiles and criterion variables were examined with the BCH command (Asparouhov and Muthén, 2014a) to test equality of means across profiles on criterion variables.

### Data preparation

Table 1 shows descriptive statistics for self-management subscales and recovery indicators. As shown in this table, only a small proportion of missing values were observed for these variables (between 0.0 and 2.0%). The same was found for background characteristics (between 0.0 and 4.7%) as well as criterion variables (between 0.0 and 0.7%). For deriving the latent profiles, which was the analysis at the core of the study, models were estimated in Mplus using a full information maximum likelihood (FIML) algorithm. This estimation method does not require deletion of cases with missing data but instead uses the information available from all the participants (Schlomer et al., 2010). This algorithm has proved to be the most robust approach for dealing with missing values without deleting cases (Newman, 2014). For the analysis performed in SPSS, deletion of cases with missing values was used. This deletion should have a negligible impact, given the very low percentage (< 5%) of missing values (De Vaus, 2002; Tabachnick and Fidell, 2013).

**Table 1:** **Correlations between the main study variables and descriptive statistics (*N* = 146–149)**.

**Variables**	***r*** **(95% CI)^a^**					
	**1**.	**2**.	**3**.	**4**.	**5**.	**6**.
1. Clinical self-management	–					
2. Empowerment self-management	0.15^t^ (−0.02, 0.31)	–				
3. Vitality self-management	0.16^t^ (−0.02, 0.31)	0.37^***^ (0.21, 0.52)	–			
4. Depression symptom severity	0.21^**^ (0.08, 0.34)	−0.34^***^ (−0.48, −0.19)	−0.30^***^ (−0.45, −0.17)	–		
5. Anxiety symptom severity	0.20^*^ (0.01, 0.35)	−0.21^**^ (−0.38, −0.03)	−0.23^**^ (−0.40, −0.06)	0.70^***^ (0.61, 0.78)	–	
6. Positive mental health	−0.03 (−0.16, 12)	0.59^***^ (0.46, 0.69)	0.41^***^ (0.24, 0.54)	−0.65^***^ (−0.74, −0.56)	−0.45^***^ (−0.58, −0.30)	–
*M*	2.32	2.39	2.10	7.70	5.64	2.65
*S.D*.	0.85	0.68	0.88	5.47	4.40	1.03
Skewness	−0.41	0.14	0.18	0.34	0.40	−0.04
Kurtosis	−0.18	−0.63	−0.68	−1.05	−1.08	−0.90
Missing	0.00%	2.01%	0.67%	0.00%	0.00%	0.00%

*Total sample size varies between 146 and 149 due to missing data on some variables*.

a *Bias-corrected accelerated confidence intervals based on N = 1000 bootstrap samples*.

*** *p ≤ 0.001*,

** *p ≤ 0.01*,

* *p ≤ 0.05*,

t *p ≤ 0.10*.

## Results

### Exploring the overall bivariate relationships of self-management and recovery indicators

As shown in Table 1, the three types of self-management strategies were positively related (correlations either significant or marginally significant). With regard to the recovery indicators, positive mental health had a negative relationship with both depression and anxiety symptom severity. Supporting the discriminant validity of the measures, the confidence interval of the correlation coefficients of positive mental health with depression and anxiety symptom severity did not include 1 (Cheng, 2011). The same observation applied for depression and anxiety symptom severity, which were positively related, but the confidence interval also did not include 1. As for the association between self-management strategies and recovery indicators, clinical strategies were positively related to depression and anxiety symptom severity, but not to positive mental health. Empowerment and vitality strategies were both negatively associated with depression and anxiety symptom severity and positively associated with positive mental health.

### Identifying the number of latent profiles and drawing their general portrait

LPA was performed using clinical, empowerment, and vitality self-management strategies, as well as depression severity, anxiety severity, and positive mental health as recovery indicators. The analysis was performed multiple times, each time increasing the requested number of profiles. As shown in Table 2, in each case, all the profiles contained more than 5% of participants (Hamza and Willoughby, 2013). *P*-value of the BLRT test suggested that adding profiles was necessary up to seven profiles. Values for AIC and SSA-BIC were increasingly lower, suggesting better fit as the number of profiles increased. A graphical examination (elbow plot, Morin, 2016) of the evolution of these indicators showed that the slope flattened after four profiles (with only minimal decrease with more profiles subsequently). BIC and CAIC were lowest for the four-profile model. However, according to the VLMR and ALMR, models with more than three profiles were not necessary. Given this pattern of indices, the three-profile and four-profile models were both examined. Three profiles from these two models showed a very similar pattern in terms of self-management and recovery indicators. The only difference was the fourth profile of the four-profile model. This profile did not add substantive meaning (i.e., scores were moderate on all indicators, which is not particularly relevant in terms of Provencher and Keyes' theory). For the sake of parsimony and because of its greater theoretical conformity, the three-profile model was thus selected as the final one. The entropy value was high. Table 3 shows the classification quality was satisfactory, with high probabilities of participants' belonging in the assigned profile (between 0.92 and 0.97) and low cross-probabilities (between 0.01 and 0.07).

**Table 2:** **Fit of the compared latent profile models with increasing numbers of profiles (*N* = 149)**.

**Number of profiles (*k*)**	**LL**	**FP**	**AIC**	**BIC**	**CAIC**	**SSA-BIC**	***P*-value VLMR**	***P*-value ALMR**	***P*-value BLRT**	**Entropy**	** < 5% of sample**
1	−1638.53	12	3301.07	3337.11	3349.11	3299.14	–	–	–	–	No
2	−1532.69	19	3103.39	3160.46	3179.46	3100.33	0.000	0.000	0.000	0.87	No
3	−1507.23	26	3066.45	3144.56	3170.56	3062.27	0.020	0.022	0.000	0.90	No
4	−1484.83	33	3035.66	3134.79	3167.79	3030.35	0.176	0.186	0.000	0.85	No
5	−1472.26	40	3024.52	3144.67	3184.67	3018.08	0.424	0.433	0.010	0.86	No
6	−1457.29	47	3008.58	3149.77	3196.77	3001.03	0.382	0.387	0.000	0.87	No
7	−1445.78	54	2999.56	3161.77	3215.77	2990.88	0.463	0.469	0.030	0.88	No
8	−1433.93	61	2989.86	3173.10	3234.10	2980.05	0.305	0.309	0.070	0.89	No

*LL, loglikelihood; FP, number of free parameters; AIC, Akaike Information Criteria; BIC, Bayesian Information Criteria; CAIC, Consistent AIC; SSA-BIC, Sample-Size-Adjusted BIC; VLMR, Vuong-Lo-Mendell-Rubin Likelihood Ratio Test for k-1 profiles vs. k profiles; ALMR, Adjusted Lo-Mendell-Rubin Likelihood Ratio Test for k-1 profiles vs. k profiles; BLRT, Bootstrapped Likelihood Ratio Test for k-1 profiles vs. k profiles*.

**Table 3:** **Average latent profile probabilities for most likely latent profile membership (row) by latent profile (column) (*N* = 149)**.

	**Profile 1: Floundering**	**Profile 2: Struggling**	**Profile 3: Flourishing**
Profile 1: Floundering	0.952	0.024	0.024
Profile 2: Struggling	0.070	0.921	0.009
Profile 3: Flourishing	0.021	0.007	0.972

**Figure 1:**
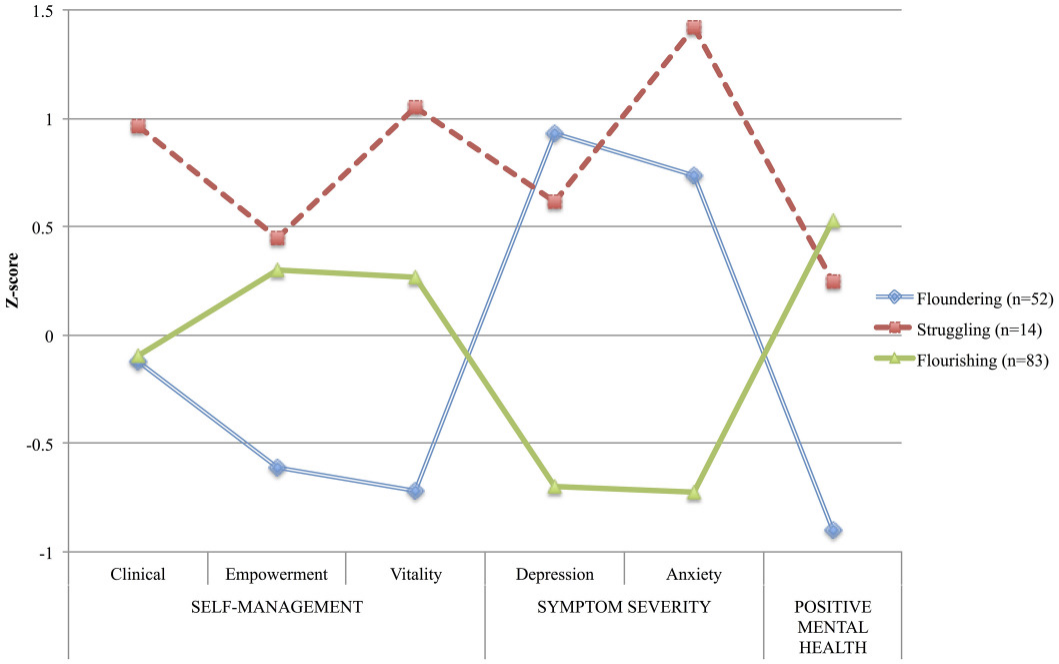
**Plot of the standardized means of the latent profiles on indicators (*N* = 149) compared to the overall sample mean**.

Figure 1 shows the standardized means of participants in each profile on the variables used in the LPA. Table 4 presents results from the equality of means and chi-square tests comparing the profiles to one another on these variables. Based on the overall pattern of these results, a summary label inspired by Keyes and Lopez's (2002) classification was assigned to each profile, which admittedly could not fully convey, in just a few words, the recovery dynamics underlying each profile.

**Table 4:** **Comparison of the latent profiles on the profile variables (continuous) and their dichotomized version**.

**Equality of means results^a^**					**Chi-square results^b^**				
**Continuous indicators**	**Floundering *M* (*S.E*.)**	**Struggling *M* (*S.E*.)**	**Flourishing *M* (*S.E*.)**	**χ^2^**	**Dichotomized indicators**	**Floundering *n* (*%*)**	**Struggling *n* (*%*)**	**Flourishing *n* (*%*)**	**χ^2^**
**SELF-MANAGEMENT**^*c*^									
Clinical	2.21_a_ (0.11)	**3.12**_b_ **(0.19)**	2.24_a_ (0.10)	24.43^***^	Score ≥ 3 (*strategies used often)*	8_a_ (15.4)	**10**_b_ **(71.4)**	18_a_ (21.7)	19.53^***^
Empowerment	1.98_a_ (0.08)	**2.70**_b_ **(0.18)**	**2.59**_b_ **(0.07)**	31.38^***^	Score ≥ 3 (*strategies used often)*	3_a_ (5.9)	**6**_b_ **(46.2)**	**26**_b_ **(31.7)**	15.36^***^
Vitality	1.47_a_ (0.10)	**3.03**_b_ **(0.20)**	2.33_c_ (0.09)	66.84^***^	Score ≥ 3 (*strategies used often)*	2_a_ (3.8)	**9**_b_**(64.3)**	25_c_ (30.5)	25.68^***^
**RECOVERY INDICATORS**									
Depression^d^	**12.77**_a_ **(0.54)**	**11.06**_a_ **(1.19)**	3.89_b_ (0.35)	172.61^***^	Score ≥ 10 (clinical cut-off)	31_a_ (59.6)	**14**_b_ **(100.0)**	2_c_ (2.4)	81.99^***^
Anxiety^e^	8.86_a_ (0.44)	**11.89**_b_ **(0.57)**	2.47_c_ (0.26)	311.56^***^	Score ≥ 8 (clinical cut-off)	**41**_a_ **(78.8)**	**8**_a_ **(57.1)**	2_b_ (2.4)	86.58^***^
Positive mental health^f^	1.73_a_ (0.10)	**2.90**_b_ **(0.22)**	**3.20**_b_ **(0.09)**	102.92^***^	Score > 3 (*positive manifestations about 2 or 3 times/week*)	1_a_ (1.9)	**5**_b_ **(35.7)**	**54**_b_ **(65.1)**	53.12^***^

*Total sample size varies between 146 and 149 due to missing data on some of the variables*.

a *For each indicator, means with different subscripts are different at p ≤ 0.05 according to equality of means results, and cells in bold highlight the profiles with the highest average scores*.

b *Percentages calculated on non-missing data. For each indicator, proportions with different subscripts are different at p ≤ 0.05 according to post-hoc tests (Bonferroni), and cells in bold highlight the profiles with the highest proportions*.

c *Measured with the Mental Health Self-management Questionnaire, scores from 0 (Never) to 4 (Very often)*.

d *Measured with the Patient Health Questionnaire 9, scores from 0 (None) to 27 (Severe)*.

e *Measured with the General Anxiety Disorder 7, scores from 0 (None) to 21 (Severe)*.

f *Measured with the Mental Health Continuum–Short Form, scores from 0 (Never) to 5 (Every day)*.

*** *p ≤ 0.001*.

The first profile—those who were *Floundering*, yet trying to manage their symptoms—included 52 participants (34.9%). These had moderately severe depression and anxiety symptoms, as well as the lowest level of positive mental health among the three profiles. More than half scored over the clinical cut-off for moderate depression and anxiety symptoms, and only one participant had a high level of positive mental health. Their use of self-management strategies was overall low to moderate, and empowerment and vitality strategies were used significantly less often than in the other profiles. Less than 10% of participants in this profile used these strategies often or very often. The second profile—*Struggling*, but fully engaged—was comprised of 14 participants (9.4%) and included those who, overall, performed self-management strategies often, and more frequently than those in other profiles for clinical and vitality strategies. Their use of vitality strategies was more than one SD above the overall sample mean. All participants in this profile scored above the clinical cut-off for depression symptom severity. They also reported a higher level of anxiety symptoms compared to the overall sample (more than one SD above the mean). Despite this pattern of symptoms similar to the *Floundering* profile, participants from the *Struggling* profile reported experiencing a higher positive mental health level. The last profile—those well on the way to *Flourishing*—was the most frequent (*n* = 83, 55.7%) and included participants with relatively high levels of self-management, especially empowerment. They had the least severe symptoms of depression and anxiety (< 3% above the clinical-cut off) compared to other profiles, as well as a high level of positive mental health (65% had high scores).

### Describing the specific self-management strategies used in each profile

Profiles were compared regarding use of the 18 specific self-management strategies measured in the questionnaire. As shown in Table 5, only two self-management strategies were used to the same extent by people in the different profiles: participating in a support or help group (low frequency) and taking medication for one's mental health problem (high frequency). The remaining clinical strategies (looking for available help resources, consulting a professional, and being actively involved in one's follow-up with professionals) were used more frequently, between *often* and very *often*, by people in the *Struggling* profile as compared to the two other profiles. Overall, empowerment strategies were used between *very rarely* or *sometimes* by people in the *Floundering* profile. In contrast, as a general pattern, participants in the *Struggling* and *Flourishing* profiles used these strategies between *sometimes* and *often*. These two profiles used the following empowerment strategies more frequently, compared to *Floundering* participants: trying to solve one's problem one step at a time, trying to recognize relapse signs, focusing one's attention on the present moment, learning to live with one's strengths and weaknesses, trying to love oneself, and finding comfort in people around oneself. Finally, participants in the *Struggling* profile used all the vitality strategies more frequently (overall between *often* and *very often*) than those in the other profiles: doing activities one enjoys to maintain a healthy lifestyle, engaging in sports, having healthy eating habits, and doing relaxation exercises.

**Table 5:** **Comparisons of latent profiles on the frequency of use of self-management strategies**.

**Items from the Mental Health Self-management Questionnaire**	**Floundering *M (S.E.)***	**Struggling *M (S.E.)***	**Flourishing *M (S.E.)***	**χ^2^**
**CLINICAL SELF-MANAGEMENT**				
I look for available resources to help me with my difficulties (websites, organizations, healthcare professionals, books, etc.).	2.23_a_ (0.16)	**3.69**_*b*_ **(0.19)**	2.42_a_ (0.13)	39.23^***^
I consult with a professional (doctor, psychologist, social worker, etc.) concerning my mental health disorder.	2.59_a_ (0.18)	**3.43**_*b*_ **(0.30)**	2.19_a_ (0.16)	13.41^***^
I get actively involved in my follow-up with the healthcare professionals I consult (physician, psychologist, social worker, etc.).	2.22_a_ (0.18)	**4.00**_*b*_ **(0.11)**	2.45_a_ (0.16)	96.12^***^
I participate in a support or help group to help me manage my difficulties.	0.59 (0.15)	1.71 (0.51)	0.68 (0.13)	4.19 n.s.
I take medication for my mental health problem as directed by a healthcare professional.	3.12 (0.22)	3.45 (0.33)	3.50 (0.13)	2.26 n.s.
**EMPOWERMENT SELF-MANAGEMENT**				
I try to solve my problems one step at a time.	2.13_a_ (0.14)	**2.84**_*b*_ **(0.27)**	**2.52**_*b*_ **(0.11)**	6.73^*^
I try to recognize the warning signs of a relapse of my mental health disorder.	2.40_a_ (0.13)	**3.05**_*b*_ **(0.26)**	**3.01**_*b*_ **(0.11)**	12.77^**^
I learn to differentiate between my mental health problem and myself as a person.	1.83_a_ (0.15)	2.01_a, b_ (0.27)	**2.50**_*b*_ **(0.14)**	10.56^**^
I focus my attention on the present moment.	1.83_a_ (0.14)	**2.47**_*b*_ **(0.26)**	**2.72**_*b*_ **(0.11)**	25.03^***^
I learn to live with my strengths and weaknesses.	2.08_a_ (0.13)	**3.08**_*b*_ **(0.21)**	**2.82**_*b*_ **(0.10)**	23.18^***^
I congratulate myself for my successes, large and small.	1.73_a_ (0.16)	2.44_a, b_ (0.35)	**2.43**_*b*_ **(0.13)**	11.83^**^
I try to love myself as I am.	1.73_a_ (0.13)	**2.57**_*b*_ **(0.28)**	**2.65**_*b*_ **(0.11)**	30.41^***^
I take my capabilities into account when arranging my schedule.	1.97_a_ (0.17)	2.67_a, b_ (0.41)	**2.42**_*b*_ **(0.12)**	5.19^*t*^
I find comfort, I feel listened by people around me.	1.92_a_ (0.14)	**3.01**_*b*_ **(0.32)**	**2.42**_*b*_ **(0.12)**	12.06^**^
**VITALITY SELF-MANAGEMENT**				
I do activities I like to maintain an active lifestyle.	1.36_a_ (0.12)	**3.31**_*b*_ **(0.27)**	2.49_c_ (0.12)	64.43^***^
I engage in sport, physical activity.	1.02_a_ (0.15)	**3.41**_*b*_ **(0.22)**	2.19_c_ (0.15)	84.06^***^
I have healthy eating habits.	2.06_a_ (0.13)	**3.54**_*b*_ **(0.19)**	2.99_c_ (0.10)	49.51^***^
I do exercises to relax (yoga, tai chi, breathing techniques, etc.).	1.12_a_ (0.15)	**2.85**_*b*_ **(0.34)**	1.72_c_ (0.14)	22.05^***^

*Response scale: 0 (Never), 1 (Very rarely), 2 (Sometimes), 3 (Often), and 4 (Very often). Items were presented to participants in French. The English version above was produced using a back-translation approach (Vallerand, 1989)*.

*Total sample size varies between 142 and 149 due to missing data on some of the items. In each line, means with different subscripts are different at p ≤ 0.05, and cells in bold highlight the profiles with the highest average scores*.

*** *p ≤ 0.001*,

** *p ≤ 0.01*,

* *p ≤ 0.05*,

t *p ≤ 0.10*.

### Characterizing the participants in each latent profile

Table 6 presents the profiles' associations with the participants' background characteristics. Probability of self-reporting a depression diagnosis was higher for the *Floundering* or *Struggling* profiles than for the *Flourishing* profile. Probability of self-reporting an anxiety disorder diagnosis was higher for the *Floundering* profile than for the *Flourishing* profile. Probability of self-reporting a bipolar disorder diagnosis was higher for the *Flourishing* profile than for the *Floundering* profile. Being currently involved in psychotherapy was more likely for the *Struggling* profile than for the two other profiles. Probability of being a man was higher in the *Floundering* profile than the other two profiles. Probability of living in a low-income household or probability of being single were higher for the *Floundering* profile than for the *Flourishing* profile.

**Table 6:** **Associations between participants' background characteristics and latent profiles**.

**Characteristics**	**Estimated probability of each characteristic within each profile**			**χ^2^**
	**Floundering**	**Struggling**	**Flourishing**	
**SELF-REPORTED DIAGNOSIS**				
Depressive disorder	**0.69**_*a*_	**0.83**_*a*_	0.43_b_	13.24^***^
Anxiety disorder	**0.53**_*a*_	0.28_a, b_	0.29_b_	6.61^*^
Bipolar disorder	0.21_a_	0.30_a, b_	**0.47**_*b*_	10.32^**^
Comorbidity between depressive, anxiety and/or bipolar disorders	0.34	0.43	0.19	4.78^*t*^
**SELF-REPORTED TREATMENTS**				
Pharmacotherapy in the last month	0.81	0.81	0.89	1.65 n.s.
Current psychotherapy	0.43_a_	**0.90**_*b*_	0.28_a_	26.17^***^
**SOCIODEMOGRAPHIC VARIABLES**				
Age	*M* = 40.11; *S.E*. = 1.90	*M* = 44.61; *S.E*. = 3.26	*M* = 41.77; *S.E*. = 1.36	1.37 n.s.
Gender (man vs. woman)	**0.31**_*a*_	0.06_*b*_	0.15_*b*_	5.07^*t*^
Education level (university vs. lower)	0.53	0.54	0.67	2.40 n.s.
Low income (yes vs. no)	**0.39**_*a*_	0.15_a, b_	0.14_b_	7.45^*^
Single (yes vs. no)	**0.76**_*a*_	0.43_a, b_	0.39_b_	16.04^***^

*For the low-income variable, only participants from Canada were included, given that this variable was created only for this subgroup. Thus, probabilities were calculated on available data (total sample size varies between 135 and 149 depending on the characteristic considered). In case of a significant chi-square, for each indicator, probabilities with different subscripts are different at p ≤ 0.05, and the cell in bold highlights the profile with the highest probability*.

*** *p ≤ 0.001*,

** *p ≤ 0.01*,

* *p ≤ 0.05*,

t *p ≤ 0.10*.

### Verifying the associations of profiles with criterion variables

As shown in Table 7, people in the *Struggling* and *Flourishing* profiles appraised their personal goals more positively and reported participating more frequently in society, compared to those in the *Floundering* profile. They also reported having more developed self-care abilities and using more adaptive coping (behavioral and cognitive) to deal with the stress associated with their mental health problem. This is consistent with these people's higher levels of positive mental health and engagement in self-management strategies. Also converging with the fact that the highest level of self-management was found in the *Struggling* profile, this profile had among the highest scores for all coping types. Interestingly, the *Struggling* and the *Floundering* profiles scored as high for avoidance coping. Their scores indicated a relatively low frequency of this type of coping, but nevertheless higher than in the *Flourishing* profile. This shared aspect of the *Floundering* and *Struggling* profiles, in terms of the use of this maladaptive coping style, is consistent with the fact that both profiles presented more severe symptoms.

**Table 7:** **Comparisons of latent profiles on criterion variables**.

**Criterion variables**	**Floundering *M (S.E.)***	**Struggling *M (S.E.)***	**Flourishing *M (S.E.)***	**χ^2^**
Personal goal appraisal^a^	4.58_a_ (0.25)	**6.99**_*b*_ **(0.37)**	**7.16**_*b*_ **(0.14)**	80.01^***^
Social participation^b^	1.00_a_ (0.06)	**1.71**_*b*_ **(0.18)**	**1.50**_*b*_ **(0.06)**	37.16^***^
Self-care abilities^c^	3.50_a_ (0.11)	**4.17**_*b*_ **(0.14)**	**4.41**_*b*_ **(0.06)**	55.28^***^
Emotional coping^d^	1.86_a_ (0.10)	**2.59**_*b*_ **(0.15)**	2.13_c_ (0.08)	15.66^***^
Behavioral coping^d^	1.47_a_ (0.10)	**2.32**_*b*_ **(0.18)**	**2.12**_*b*_ **(0.08)**	29.86^***^
Cognitive coping^d^	0.88_a_ (0.07)	**1.45**_*b*_ **(0.07)**	**1.50**_*b*_ **(0.06)**	51.12^***^
Avoidance coping^d^	**1.02**_*a*_ **(0.07)**	**0.94**_*a*_ **(0.13)**	0.66_b_ (0.06)	16.41^***^

*Total sample size varies between 148 and 149 due to missing data on a criterion variable. In each line, means with different subscripts are different at p ≤ 0.05, and cells in bold highlight the profiles with the highest average scores*.

a *Measured with the Personal Project System Rating Scale, scores from 1 (Very negative) to 10 (Very positive)*.

b *Measured with the Social Participation Scale, scores from 0 (Never) to 4 (Almost every day)*.

c *Measured with the Therapeutic Self-Care Scale, scores from 0 (Not at all) to 5 (Completely)*.

d *Measured with the Brief COPE, scores from 0 (Not at all) to 3 (A lot)*.

*** *p ≤ 0.001*.

## Discussion

In line with the shift of mental health services toward a person-centered approach (Corrigan, 2015), the present study explored for the first time individual recovery profiles. The results suggest three such profiles underlying the engagement of people with mental disorders in their recovery. Their pattern of associations with criterion variables (personal goal appraisal, social participation, self-care abilities, coping) was consistent with previous theoretical and empirical work on factors that form the foundation of successful self-management and mental health recovery. In keeping with the description of these profiles in terms of recovery indicators and self-management strategies, the *Floundering* profile presented the most unfavorable portrait on the criterion variables, while the *Flourishing* profile presented the most favorable portrait, and the in-between *Struggling* profile presented a mostly favorable, yet mixed portrait.

### Understanding self-management differently

Although traditional variable-oriented analytical strategies are useful for seeing the big picture of how specific variables relate to each other at the group level, they are insufficient to inform health professionals working from a person-centered perspective (Cloninger, 2013). In contrast, there is a natural fit between the person-centered philosophy of care and person-oriented statistical analysis, because both recognize the person as more than the sum of parts (Laursen, 2015). Nevertheless, person-oriented analysis is still rarely used even to study topics closely related to person-centered care, such as people's engagement in self-management and recovery. Our study illustrates that person-oriented analysis can provide insightful results with the potential to stimulate reflection.

By definition, from a traditional variable-oriented perspective, positive associations would have been expected between self-management and recovery. By extension, it would have been expected that those who were more engaged in strategies to reduce their symptoms (clinical self-management), trying more actively to gain control by harnessing their positive sense of self (empowerment self-management), and adopting a healthier and active lifestyle (vitality self-management) would have had less severe symptoms as well as higher levels of positive mental health. Of the three identified profiles, the *Floundering* and *Flourishing* profiles were overall in line with this reasoning. Participants in the *Floundering* profile used empowerment and vitality self-management strategies less frequently than did those who were *Flourishing*. As a corollary, people in the former profile scored more negatively on recovery indicators than did those in the latter profile. However, despite their different scores on recovery indicators, people in both profiles reported using clinical strategies to the same extent (only moderately) as part of their self-management “recipe.” This provides evidence that the relationship between self-management and recovery indicators is not as straightforward as might be thought, at least when studied from a cross-sectional perspective.

In that same vein, a surprising result was seen in the *Struggling* profile, where respondents reported high self-management co-existing with moderately severe symptoms. People in this profile were the most activated and were involved in a diverse combination of frequently used clinical, empowerment, and vitality self-management strategies. They were also more likely to be currently involved in psychotherapy, potentially indicating or resulting from their higher engagement (see review from Kreyenbuhl et al., 2009, on engagement and treatment). Their symptoms were among the most severe observed across the different profiles, suggesting that a high level of engagement, even in clinical strategies specifically targeting symptoms, is not necessarily associated with reduced symptomatology. Indeed, these participants had on average the most severe anxiety levels and used avoidance coping strategies (i.e., substance use, denial, behavioral disengagement) to the same extent as did *Floundering* participants. Even though *Struggling* participants' score on the use of such maladaptive coping strategies was low, it was nevertheless similar to levels observed in studies with other clinical samples (Meyer, 2001; Nazir and Mohsin, 2013). One of those studies (Meyer, 2001) suggested that the use of maladaptive coping is associated with higher symptom severity. A review of the literature supports the notion that avoidance coping could be associated with relapse, recurrence, and greater time to recovery in mood disorders (Christensen and Kessing, 2005). Over the long term, use of avoidance coping has been shown to generate stress, which can increase symptoms (Holahan et al., 2005).

It is also possible that *Struggling* participants' focus on working through their symptoms elevated their stress level. This would be consistent with literature suggesting that, as part of the health engagement process, people with chronic diseases tend to experience a phase of arousal in which they are hyper-attentive to their symptoms yet are still unable to cope adequately, causing them anxiety (Barello et al., 2014; Graffigna et al., 2014). Taking part in a psychotherapeutic process can also be demanding for a person, especially when using stressful procedures such as exposure (Wills, 2008). The burden associated with self-management can also cause stress (Sav et al., 2013). Seeking to improve one's happiness has been shown to be “a delicate art” that can backfire (Catalino et al., 2014, p. 1160). Likewise, our cross-sectional results may suggest that actively seeking to get better and wanting to do the best for one's health might put additional stress on people with mood and anxiety disorder, at least temporarily or in the short term.

An alternative interpretation is that participants in the *Struggling* profile engaged in self-management to deal with their residual symptoms. The literature on depression (the diagnosis most reported in this profile) is clear on the fact that, even when responding successfully to pharmacotherapy or psychotherapy, a significant proportion of people still have to contend with incapacitating residual symptoms (see review from Fava et al., 2007, and by Nierenberg, 2015). Anxiety is one of the most common residual symptoms in depression disorders (Fava et al., 2007; D'Avanzato et al., 2013). From that standpoint, it is possible that *Struggling* participants' symptoms (notably their relatively high anxiety) did not result from their active self-management, but rather were the very reason why they actively engaged in self-management. These participants' attempts to deal with stressful residual symptoms may also explain their involvement in a diversity of coping strategies, as shown by their elevated coping scores, even on apparently contradictory subscales (e.g., avoidance vs. behavioral coping). As put forward by Folkman and Lazarus (1991), a person may seek and try several, sometimes opposite, ways of dealing with a stressful situation. While persons in this profile may not be reaping the benefits of their coping and self-management efforts in the moment, they might experience less severe symptoms over the longer term. Longitudinal studies exploring how symptom severity and self-management relate to each other over time are needed to verify this.

### Supporting and expanding the complete mental health recovery model

Provencher and Keyes's Complete Mental Health Recovery model (2010, 2011, 2013) was developed on the idea that symptom severity and positive mental health are two distinct dimensions and that their intersections form six states of recovery. This proposition was based on studies in which participants from the general population were artificially classified into different profiles corresponding to these six states (Keyes, 2005, 2007). Our results based on an inductive method of classification (LPA) confirm the existence of some of these profiles, thereby providing general supporting evidence for their model.

The *Flourishing* profile found in the present study resembles the state described by Provencher and Keyes (2010, 2011, 2013) in which the person is recovered in terms of symptom severity and shows a moderately high level of positive mental health. Similarly, the *Floundering* profile mirrors their description of the opposite state (non-recovered from the mental illness and low positive mental health). Finally, the *Struggling* profile echoes Provencher and Keyes' (2010, 2011, 2013) state of non-recovery from symptoms concomitant with a moderate level of positive mental health. Although our participants were not numerous in this profile, its existence is supported by the model's adequate fit and the satisfactory classification probabilities. The existence of this profile is essential because it demonstrates the foundational idea that people with important mental health symptoms can nevertheless experience frequent manifestations of well-being that help make their life worth living, as positive psychologists would say (Seligman et al., 2004). Three others states (e.g., recovered from mental illness and low positive mental health) proposed by Provencher and Keyes (2010, 2011, 2013) were not found in the present study. However, it is possible that, with a larger sample size, probabilities of observing these would have been augmented. Even in the large general population studies cited above (Keyes, 2005, 2007), such states have been shown to be among the least frequent.

Beyond providing confirmation, the present study complements the Complete Mental Health Recovery model by explicitly incorporating self-management strategies. Provencher and Keyes (2010) recognized people's active role in their recovery and gave examples of strategies that could promote the process. The present study expands on this by providing unprecedented empirical data on the level of self-management engagement shown by people in different profiles of recovery. It also reveals specific self-management strategies that people in each profile tend to combine.

The level of engagement in almost all self-management strategies was lowest for participants in the *Floundering* profile. Although time since onset of their disorder was not collected, this profile relates to the description of people who are in the beginning of the recovery process (Provencher and Keyes, 2010). Researchers have labeled this the “moratorium” stage, characterized by hopelessness and self-protective withdrawal (Andresen et al., 2003, 2006). Taking their medication as prescribed was the only self-management strategy that participants from this profile implemented on a regular basis, which seems consistent with the dependence on external support that distinguishes this beginning stage (Andresen et al., 2006).

People in the *Struggling* profile had the highest level of self-management. Their combination of self-reported strategies was characterized by regular use of help-seeking strategies (e.g., inform oneself about resources, consult with a professional), in line with their higher probability of being involved in psychotherapy and having more severe symptoms (Hämäläinen et al., 2008). They also were keeping themselves physically active and healthy by maintaining a good diet and engaging in sports and relaxation exercises. Among other strategies, they were trying to solve their problems one step at a time and to focus on the present moment. These self-management strategies evoke lifestyles changes, behavioral activation, problem resolution, and mindfulness activities that are suggested or recommended in clinical guidelines (e.g., National Institute for Health and Care Excellence, 2009; Scottish Intercollegiate Guidelines Network, 2010). Participants in this profile may have been encouraged to use such strategies by a psychotherapist or other health professional they consulted. Given their use of potentially physically energizing strategies, it is not surprising that their level of positive mental health was relatively high, in keeping with a recent qualitative study showing a sense of energy to be a marker of positive mental health in people with mental disorders (Mjøsund et al., 2015). Although such a profile of self-management strategies has not been described explicitly in the literature before, it bears some resemblance to descriptions of recovery stages after the initial “moratorium” (Andresen et al., 2003). In those stages the individual struggles with the illness but, at some turning point, manages to move into action (Davidson and Strauss, 1992; Spaniol and Wewiorski, 2012).

As for those in the *Flourishing* profile, their moderately high self-management scores suggested that, although well on the way to full recovery, they were still very engaged in getting better. Even though taking their medication as prescribed and recognizing relapse signs were important for them, in all likelihood their main focus was not on managing the disorder for itself, but rather for the benefit of optimizing their overall well-being. Provencher and Keyes (2011, p. 64), described people at similar states of recovery: “They look for opportunities to challenge themselves and to reach a sense of serenity and peace of mind. […] When deficits are still present, individuals are well aware of them and know how to best use them while continuing to grow and to optimize their own potential in the pursuit of challenging goals.” Consistent with this portrayal, the strategies characteristic of the *Flourishing* profile were related to accepting, working around, and transcending difficulties, such as arranging their schedule around their capabilities and congratulating themselves on their successes. This pattern of self-management strategies is consistent with the final stages of the recovery process (“rebuilding” and “growth”), in which people forge a new positive sense of self and develop a feeling of confidence in their abilities to face challenges (Andresen et al., 2003, 2006).

### Bringing background characteristics and recovery inequalities to the foreground

Guidelines for person-centered health services emphasize the importance of culturally sensitive assessment and intervention practices (Adams et al., 2004; Porche, 2013) that are tailored or individualized to the person's background (Lauver et al., 2002). The present study revealed several background characteristics associated with each profile. Most notably, the least favorable profile (*Floundering*) was characterized by an array of clinical (self-reported depressive or anxiety disorder) and sociodemographic variables (male gender, low income, and singlehood). In contrast, the most favorable profile (*Flourishin*g) was characterized by a different clinical background (self-reported bipolar disorder), as well as the opposite sociodemographic variables (being a female, having sufficient income, and having a life partner). These variables may represent risk and protective factors for practitioners to consider in their holistic comprehension of their clients' situation.

Consistent with a previous study (Vermeulen-Smit et al., 2015) suggesting that anxiety disorders could be associated with a form of unhealthy lifestyle, the *Floundering* profile was the profile most clearly characterized by an overrepresentation of people with a self-reported anxiety disorder, and was the least engaged in vitality self-management strategies. Also of particular interest was the association of the *Floundering* profile with social variables (gender, singlehood, low income), in line with several previous studies in the wider mental health field. For example, several studies have shown singlehood to be related to higher prevalence of depression and anxiety (see Martins et al., 2012). In a recent study of people with a depressive disorder, single marital status at baseline predicted non-recovery in terms of depressive symptoms 11 years later (Markkula et al., 2016), which is congruent with a stream of research concerning the association of marital status with health and health behaviors. This relation could be due to multiple reasons, such as the fact that economic, psychological, and social resources are less accessible to single people (see reviews from Robards et al., 2012; Robles et al., 2014). Economic disadvantage is also associated with higher prevalence of depression and anxiety disorders (see Martins et al., 2012). It has been suggested that psychosocial resources helpful for coping effectively with life stressors, such as personal control and social support, may be less available to disadvantaged people (Taylor and Seeman, 1999). People with low incomes are also more likely to face financial barriers to obtaining mental health services (Sareen et al., 2007).

Concerning gender, although anxiety and mood disorders prevalence rates are generally higher in women than in men (Faravelli et al., 2013; see reviews from Piccinelli and Wilkinson, 2000; Bekker and van Mens-Verhulst, 2007), research has documented several health challenges faced by men, such as lower subjective well-being (Graham and Chattopadhyay, 2013) and higher suicide rates (Nock et al., 2008). Men also tend to have less healthy lifestyles (Von Bothmer and Fridlund, 2005) and to consult less than women in cases of emotional problems, due to their endorsement of traditionally masculine cultural norms (Möller-Leimkühler, 2002). Overall results from the present study expand these previous findings by pointing out potential social inequalities in terms of chances of recovery from mood and anxiety disorders.

### Implications for patient-centered interventions

From a person-centered care perspective, people's idiosyncratic recovery profiles (in terms of self-management strategies and recovery indicators) should be considered by professionals who intervene with them. Traditional self-management support interventions usually focus on symptom reduction (e.g., Bilsker and Patterson, 2007; Lorig et al., 2014). Our findings confirmed that people use different combinations of self-management strategies, focusing not only on symptoms, but also on promoting their overall positive mental health. Thus, health professionals should consider the whole diversity of self-management behaviors implemented by their clients. Through a comprehensive investigation, professionals can seize opportunities to build clients' confidence by offering sincere praise for their self-management actions, even small ones, in line with solution-focused principles (Winbolt, 2011).

The low frequency of self-management strategies observed in the *Floundering* profile might warrant discussions with clients in such a profile to identify potential emotional (e.g., feeling of incompetence) and cognitive (e.g., lack of knowledge) barriers to self-management. Health engagement in the context of chronic illness is intertwined with emotional and cognitive processes (Graffigna and Barello, 2015; Graffigna et al., 2015a). If done appropriately and respecting the individual's wishes, working through these barriers together could help set the client on a path of increased engagement in self-management, and ultimately into the *Flourishing* profile. To that end, the recently validated Patient Health Engagement Scale (Graffigna et al., 2015a) is a 5-item short scale to help practitioners identify their clients' position in their engagement process, considering the emotional and cognitive components. Discussing this scale's results in the clinical encounter can be useful to stimulate person-centered communication between practitioner and client (Graffigna et al., 2015a). Such a client–practitioner partnership could facilitate engagement in self-management (Trivedi et al., 2007).

Results from the *Struggling* profile highlight a possibility that anxiety can arise, at least temporarily, from engaging deeply in self-management. Although the level of self-management was not sufficiently high to be deemed excessive in itself in the present study, the existence of this profile raises a yellow flag. In self-management, as in other domains of life, it is possible that excessiveness causes stress and leads to negative outcomes (Witkin, 1985). While being respectful of clients' engagement, professionals could personalize follow-ups to support people in achieving the delicate balance between actively managing their illness and pursuing other life activities and goals without undue stress.

Our findings suggest that additional efforts should be expended to ensure that mental health services effectively reach and support men, single persons, and those with low incomes in their self-management and recovery. Examples of interventions from the chronic illness or physical health field can be instructive for this purpose, such as self-management interventions developed for people on low income with diabetes (Eakin et al., 2002), or the Scottish Premier League football clubs, which promote weight reduction in men through a gender-sensitized context, content, and style of delivery (Hunt et al., 2014). In 2014, the Geneva Declaration on Person- and People-centered Integrated Health Care for All was adopted, which encouraged commitment to reducing health inequalities and to making person-centered care available for all (Cloninger et al., 2014). This requires not only adapting professional services to people's profiles, but also committing to social justice and participating in wider efforts aimed at “creating well-being-promoting societies as well as treating illness” (Slade, 2010, p. 9).

### Limitations and future research

The present study is limited by its cross-sectional design. The profiles discovered represent static “snapshots” of the recovery experience taken at one moment in time. As recovery is thought to unfold across time, with “setbacks and plateaus along the way” (Farkas, 2007, p. 72), it is possible that the different profiles are experienced at different moments in the recovery process. Capturing time elapsed since the onset of the disorder would have enabled a first examination of this question, but unfortunately it was not measured in this study. Provencher and Keyes (2011) suggested that people transition from one state to another on the pathway toward complete mental health recovery. One can intuitively conceive that the *Flourishing* profile is more likely to be experienced later in the recovery process, while the *Floundering* profile is more likely to be experienced at the beginning of the process. The *Struggling* profile might represent an intermediate state in which the person becomes deeply engaged in self-management, possibly paving the way toward flourishing. It might also be an end-state for some people who need to deal with residual symptoms over the long run. Such speculations illustrate a set of research questions that have yet to be explored with longitudinal designs.

Although the current sample size appears to be sufficient to conduct LPA according to some suggested guidelines (e.g., Formann, 1984 in Tuma and Decker, 2013; Williams and Kibowski, 2016), it remains limited in terms of generalizability. Our sample size was modest for multivariate statistics like LPA (Mueller et al., 2010), warranting further studies to replicate the findings, especially the existence of the *Struggling* profile, in which only a limited number of participants were classified. If the power was sufficient to detect meaningful differences between profiles, larger sample sizes would make it possible to verify the few associations that were only marginally significant.

Online research provides valid data (Gosling et al., 2004) and makes it possible to reach individuals who are dispersed geographically (Wright, 2006). However, future studies would benefit from using a traditional face-to-face method, allowing the use of structured clinical interviews (e.g., Structured Clinical Interview for DSM Disorders; First et al., 2002) to thoroughly measure participants' clinical symptoms. Such objective symptom assessment could help rule out alternative interpretations for the findings. In the present study, it is possible that people in the *Struggling* profile, being focused on getting better through self-management and psychotherapy, were more conscious of their symptoms and thus biased toward giving higher scores to self-reported severity measures such as the PHQ-9 and GAD-7.

Beyond background characteristics, several other variables possibly related to profiles warrant examination. Notably, while self-management refers mainly to the *actions* involved in taking care of one's health, other cognitive (e.g., knowledge about their health) and emotional variables (e.g., feelings of confidence) are also likely to be involved and should be considered as potential determinants (Graffigna and Barello, 2015; Graffigna et al., 2015a). Also, the study did not examine health professionals' (e.g., psychiatrists, psychologists, general practitioners) contribution to self-management and recovery. A recent measure such as the INSPIRE questionnaire (Williams et al., 2015) could be useful in this regard to assess the extent to which professionals support clients in their personal recovery.

### Conclusion

Mood and anxiety disorders figure among the 20 leading causes of disability worldwide (Institute for Health Metrics and Evaluation, 2013). At the heart of person-centered approaches in mental health services (Davidson et al., 2015) lies the principle that people can play an active role in dealing with such incapacitating disorders and in promoting their complete recovery. Yet systematic research-based evidence on self-management and recovery from these disorders is scarce. The present study represents a first thorough quantitative examination of recovery, combining self-management strategies used and recovery indicators.

Although the results need to be replicated, the person-oriented analyses conducted in this study yielded insights for practitioners interested in developing services that are personalized to clients' unique profiles and backgrounds. The list of profiles identified in the study is in no way definitive. Thus, we advise practitioners not to strive to classify their clients into these exact profiles. Rather, we hope the individualized person-centered approach developed in this study can encourage them to adapt their services to their clients' own profiles.

At the theoretical levels, this study integrated notions from different domains of research and interventions, such as the chronic illness, mental health, positive psychology, and patient-centered care literature. We hope the findings will stimulate reflection on how an integrative theoretical framework and innovative methods can provide original empirical information on people's health engagement and how it supports their health and well-being.

## Author contributions

SC developed the research design, coordinated data collection, performed the statistical analyses, and wrote the manuscript as part of his Ph.D. thesis. SR contributed to the design of the study and critically reviewed the paper several times. SM conducted the qualitative interviews that served as the basis for the validated self-management questionnaire, enriching the manuscript with her experience. HP took part in planning the study as a co-investigator of the larger research project. Her knowledge as a recovery expert was useful in improving the manuscript. CH is a co-investigator of the larger research project and took part in planning the study. Her expertise on self-management helped improve the manuscript. PR took part in planning the study as a co-investigator of the larger research project. As an expert in mental health services, she critically reviewed the manuscript. MP is a co-investigator in the larger research project and contributed to its planning. His contribution to the study concerned the evaluation of symptom severity. JH is the principal investigator of the larger research project of which the present study is a part. As SC's thesis advisor, JH closely supervised all research stages and critically reviewed the paper. The authors have approved the article and agree to be accountable for all aspects of the work.

## Funding

This study was supported by a grant for young investigators (Janie Houle) from the Fonds de recherche du Québec—Santé (Grant no. 22194).

### Conflict of interest statement

The authors declare that the research was conducted in the absence of any commercial or financial relationships that could be construed as a potential conflict of interest. The reviewers, ES and SB, and handling Editor declared their shared affiliation, and the handling Editor states that the process nevertheless met the standards of a fair and objective review.

## Footnotes

^1^As described by Davidson et al. (2015), person-centered services in the mental health field emerged from the patient-centered model of care in the medical domain, for which the 2001 Institute of Medicine Report made a strong case. The expression “person-centered” is preferred to “patient-centered” throughout this article, as it is more consistent with the aim of these services, i.e., recognizing the person and his/her active role beyond the “patient” status (Davidson et al., 2015). For the same reason, the word “person” (or “client”) is preferred to “patient” in the paper.

^2^See https://www.isad.org.uk; see also the journal of the association, the Journal of Affective Disorders.

^3^In addition to a measure of symptom severity, Provencher and Keyes (2010, 2011, 2013) suggest that a measure of functional impairment should also be included to assess recovery from mental illness, based on the usual practice for schizophrenia. However, in the case of mood and anxiety disorders, the focus of the present article, inclusion of such a measure is not mandatory (e.g., Goldberg et al., 2007; Radhakrishnan et al., 2013). Furthermore, a task force (Rush et al., 2006) has specifically recommended that such a measure should not be included when evaluating clinical recovery, as observed functional impairment may be unrelated to the mental illness under consideration. For this reason, the level of functional impairment is not taken into account as a recovery indicator in the present article.

^4^We freely estimated means in all profiles. We also tested models in which variance was freely estimated (Morin et al., 2011a). However, these models were not retained given that their solutions failed to be sufficiently replicated or that they converged on improper solutions (negative variance). These problems suggest that more parsimonious models (in which variance is constrained to be equal across profiles) were more appropriate (Morin et al., 2011b), as further indicated by their better fit.
